# The Prognostic Utility of Plasma NGAL Levels in ST Segment Elevation in Myocardial Infarction Patients

**DOI:** 10.1155/2020/4637043

**Published:** 2020-08-30

**Authors:** Ahmet Avci, Bahadir Ozturk, Kenan Demir, Fikret Akyürek, Bulent Behlul Altunkeser

**Affiliations:** ^1^Zonguldak Bulent Ecevit University, Faculty of Medicine Cardiology Department, Zonguldak, Turkey; ^2^Selcuk University, Faculty of Medicine Biochemistry Department, Konya, Turkey; ^3^Selcuk University, Faculty of Medicine Cardiology Department, Konya, Turkey

## Abstract

**Introduction:**

Plasma neutrophil gelatinase-associated lipocalin (NGAL) levels in acute myocardial infarction (AMI) patients are markedly higher. In addition, plasma NGAL levels were increased in patients with acute and chronic heart failure as a complication of myocardial infarction. In this study, we investigated whether there is a difference between the prognostic use of plasma NGAL levels in ST-elevation myocardial infarction (STEMI) patients with preserved and reduced left ventricular ejection fraction (LVEF).

**Methods:**

235 consecutive STEMI patients were enrolled in the study. Patients were divided into groups according to LVEF. Plasma NGAL, troponin I, creatine kinase MB (CKMB), and C-reactive protein (CRP) were measured. Finally, the study population examined with 34 reduced LVEF and 34 preserved LVEF consisted of a total of 68 patients (12 females; mean age, 61.5 ± 14.7). All patients were followed up prospectively for 6 months. This study group was divided into two subgroups as the patients who died (*n* = 14) and survived (*n* = 34), and plasma NGAL levels of the groups were compared.

**Results:**

The median of NGAL was 190.08 ng/ml. Age, troponin I, CKMB, CRP, glomerular filtration rate, and creatinine were higher in reduced LVEF groups. Plasma NGAL levels were also higher in reduced LVEF than in preserved LVEF, but statistically not significant (*p*=0.07). Plasma NGAL levels were significantly higher in death patients than in survived patients (*p* < 0.001). In ROC curve analysis, the level to detect isolated cardiovascular mortality with a sensitivity of 86% and a specificity of 77% was 190 ng/mL for NGAL.

**Conclusion:**

Plasma NGAL levels can be used to predict cardiovascular mortality in STEMI patients.

## 1. Introduction

NGAL is a secretory glycoprotein that can be detected at very low levels in physiological conditions in many human tissues, including the kidney, heart, stomach, lungs, and colon [1, 2]. It is especially well studied in the prediction of renal impairment; it is a well-known marker of renal dysfunction. In addition, there is important evidence that NGAL may also play a role in atherosclerosis [3]. NGAL has also been shown to be expressed in endothelial cells, smooth muscle cells, and macrophages in atherosclerotic plaques. The activity of matrix metalloproteinase-9 (MMP-9) is modulated through endothelial dysfunction, inflammatory processes, and matrix breakdown, and it causes the development of atherosclerotic plaque instability [4–7].

The collagen and gelatin are important for vascular integrity and stability. MMP-9 is one of the most important molecules that increase especially in sensitive plaques and is shown in atherosclerotic plaques, which play an important role in the breakdown of gelatin and collagen of the connective tissue of the artery walls [3]. MMP-9 can play a role in plaque disruption [8]. For these reasons, NGAL can play an important role in disrupting the vascular structure [3]. NGAL is also thought to play a role in cell survival, inflammation, and matrix disruption. This study aimed at identifying the relationship between short-term prognosis and serum NGAL levels in acute STEMI patients with preserved and reduced LVEF

## 2. Materials and Methods

### 2.1. Study Population

Between January 2012 and August 2013, 235 consecutive STEMI patients were enrolled in the study. STEMI was diagnosed with persistent anginal pain for 20 minutes or more and with the presence of 1 mm or more ST segment height or the presence of new left bundle branch block in 2 or more adjacent leads other than V2 to V3. For the diagnosis, ST2 elevation of 2 mm in men and 1.5 mm in women was required in leads V2 to V3. Patients with culprit lesions in the left main coronary artery, greater than 50% stenosis in the left main coronary artery, previous coronary artery bypass surgery, old myocardial infarction, borderline left ventricular systolic function (ejection fraction: >35 and <55), chronic kidney disease (serum creatinine > 1.4 mg/dl), active infection, chronic inflammatory disease, or malignancy were excluded. Finally, the studied population consisted of 68 patients (12 females (17.6%); mean age, 61.5 ± 14.7) with STEMI. The patients were divided into two groups as preserved LVEF and reduced LVEF. The first group included the patients with LVEF ≥55 (preserved LVEF group; *n* = 34). The patients with LVEF ≤35 were included in the second group (reduced group; *n* = 34). After the institutional ethics committee approved the study protocol, detailed informed consent was received from all patients.

### 2.2. Data Analysis

Venous blood samples were obtained on admission in the coronary care unit or in the emergency department and sent to a laboratory for analysis within 1 hour after collection. Plasma glucose, LDL cholesterol, HDL cholesterol, triglycerides, blood cell count, troponin I, and serum NGAL were measured. Baseline characteristics that include presence of hypertension (HT), diabetes mellitus (DM), smoking status, and family history for coronary artery disease (CAD) and lipid parameters were recorded during the direct interview with the patient. HT, DM, and family history definition was made according to current guidelines. Smoking was defined as current smoking. Electrocardiography (ECG) was recorded, and transthoracic echocardiography with the Vivid E9 system using a 1.5–4.6 MHz probe (GE Vingmed Ultrasound AS, Horten, Norway) was performed on all patients participating in the study. The modified Simpson's rule was used to measure the LVEF. All analyses were carried out by observers blinded to the clinical and laboratory data.

### 2.3. Fibrinolytic Therapy, Coronary Angiography, Primary Angioplasty, and Stenting

All patients received chewable acetylsalicylic acid (300 mg, unless contraindicated) and oral clopidogrel (300 mg or 600 mg loading dose). 30 patients were treated with fibrinolytic therapy (accelerated infusion of tissue plasminogen activator (t-PA)), and totally 40 patients were treated with angioplasty and/or stenting (38 patients primary and 2 patients rescue). In the other 28 patients who received fibrinolytic therapy, coronary angiography was performed electively. Primary percutaneous coronary intervention was performed using standard techniques and materials in accordance with current guidelines. Concomitant medical treatments with *β*-blockers, angiotensin-converting enzyme inhibitors, and statins were started according to the guidelines of the American College of Cardiology/American Heart Association.

### 2.4. Specific Laboratory Techniques

After venous blood samples were collected, all samples underwent cold centrifugation (at 4°C) at 3500 rpm for 10 min. Aliquots of sera were stored at −80°C until analysis. The longest duration for serum sample storage was 12 months. The blood samples were studied with Human NGAL Sandwich ELISA kit (NGAL, BIOPORTO Diagnostics Immunoassay kit, BioPorto Diagnostic A/S, Gentofte, Denmark) by biochemists.

### 2.5. Clinical Follow-Up and Study End Point

All the patients were prospectively followed up for 6 months, and the month 6 assessments were conducted either on-site or via telephone. The main end point evaluated in this study was cardiovascular mortality at sixth month. Cardiovascular mortality was defined as unexpected sudden death and death result of acute myocardial infarction, heart failure (HF), or arrhythmia.

## 3. Results

The clinical and biochemical properties of STEMI patients are shown in Table 1. In this study, the mean age of patients was 61.51 ± 14.72, and 17.6% of patients were female. 30 patients were treated with fibrinolytic therapy and 40 patients treated with angioplasty and/or stenting. The median of NGAL was 190.08 ng/ml.

**Figure 1:**
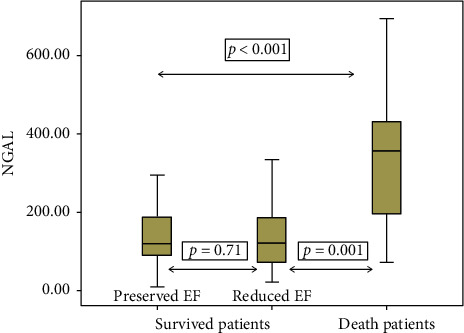
NGAL levels among study groups.

The patients were grouped according to LVEF. The first group included the patients with LVEF > 55 (preserved LVEF group; *n* = 34). The patients with LVEF < 35 were included in the second group (reduced LVEF group; *n* = 34). Age was higher in reduced LVEF groups than in preserved LVEF groups (66.79 ± 14.09 vs. 56.24 ± 13.57, *p*=0.002). Traditional cardiovascular risk factors, such as DM, HT, hyperlipidemia, smoking, and family history of CAD, have no statistically significant differences in groups. Troponin I, CKMB, CRP, glomerular filtration rate (GFR), and creatinine levels were higher in reduced LVEF groups than in preserved LVEF groups (*p*=0.008, *p*=0.002, *p* < 0.001, *p* < 0.001, and*p*=0.001, respectively). Admission times were longer in reduced LVEF groups than in preserved LVEF groups (*p*=0.01). Plasma NGAL levels were higher in reduced LVEF than in preserved LVEF, but statistically not significant (*p*=0.07) (Table 2 and Figure 1). Logistic regression analysis was performed to estimate the effects of serum NGAL levels with other cardiovascular risk factors in patients with both preserved LVEF and reduced LVEF. Reduced LVEF was associated with the age and elevated troponin level but was not associated with the serum NGAL level (Table 3).

**Figure 2:**
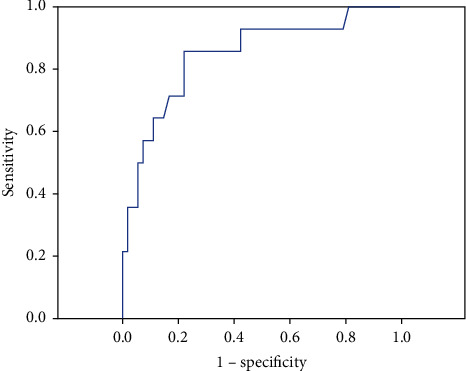
ROC curve analysis for plasma NGAL for the discrimination of death patients from survived patients with STEMI.

This study group had been further divided into two subgroups: death group (*n* = 14) and survived group (*n* = 54). Age was higher in death groups than in survived groups (73.36 ± 10.59 vs. 58.44 ± 14.14, *p* < 0.001). Hematologic, biochemical parameters, and traditional cardiovascular risk factors, such as DM, HT, hyperlipidemia, smoking, and family history of CAD, have no statistically significant differences in both groups. Admission times were longer in death groups than in survived groups (*p*=0.01). The GFR and creatinine levels were higher in death groups than in survived groups (*p* < 0.001 and *p*=0.007, respectively). Plasma NGAL levels were higher in death groups than in survived groups (357 (71–694) vs. 120 (9–513) ng/ml, *p* < 0.001)) (Table 4 and Figure 1). To investigate the effects of serum NGAL level in cardiovascular mortality together with several other cardiovascular risk factors, the logistic regression analysis was performed. Cardiovascular mortality was associated with serum NGAL levels and reduced LVEF (Table 5). In ROC curve analysis, the level to detect isolated cardiovascular mortality with a sensitivity of 86% and a specificity of 77% was 190 ng/mL. AUC was 0.845 with a 95% CI of 0.722–0.968 (Figure 2).

## 4. Discussion

In this study, we aimed to compare the plasma NGAL levels in STEMI patients with reduced LVEF and preserved LVEF. We found that STEMI patients with high NGAL levels had a higher risk of death than STEMI patients with low NGAL levels.

In this study, we found that the STEMI patients with increased NGAL levels were at much higher risk of death compared with the STEMI patients with low levels of NGAL. In addition, this study shows that for the first time in STEMI patients, NGAL's determination of cardiovascular mortality is more better than determining HF.

Previous studies attributed an important role to neutrophils and inflammation for the progression of atherosclerosis and AMI [9, 10]. Inflammation takes part in the formation and rupture of atheromatous plaques. There is substantial evidence to support the involvement of neutrophils in this inflammatory process [1]. MMPs are endopeptidases, which play a major role in atherosclerosis by degrading the extracellular matrix and causing cap rupture and intraplaque hemorrhage. MMP-9 is one of the MMPs, which are expressed in the vulnerable atherosclerotic plaque. It is therefore associated with plaque rupture and has been suggested to be involved in remodeling processes [11, 12]. NGAL is a glycoprotein found in neutrophils and responsible for the regulation of the activity of MMP-9 [13, 14]. Plasma NGAL levels are significantly elevated in epithelial damage [15]. NGAL forms a complex with MMP-9 and prevents its degradation. The increased NGAL expression was demonstrated in atherosclerotic plaques, and this leads to the enhanced proteolytic activity within the atherosclerotic plaque [1, 14].

NGAL levels increase as disease severity increases in patients with coronary artery disease [16]. Choi et al. found that the plasma NGAL level was higher in patients with CAD compared with the control group and was higher in patients with AMI compared with patients with chronic CAD [17]. In addition, Sahinarslan et al. found that the plasma NGAL level was higher in patients with AMI compared with the patients with stable CAD [16]. Lindberg et al. showed that increased plasma NGAL levels independently determined all-cause mortality and MACE in STEMI patients undergoing primary PCI [7]. In our study, the plasma NGAL level is significantly higher in died patients. This suggests that NGAL could be a prognostic marker for cardiovascular mortality in STEMI patients. Furthermore, NGAL is a novel biomarker of human kidney injury and HF.

. Many studies recommend the use of plasma NGAL levels in patients hospitalized for acute HF to estimate the risk [2]. Bolignano et al. reported that NGAL levels were significantly increased in patients with HF [18]. It is also known that serum NGAL levels were increased in acute and chronic HF patients after acute myocardial infarction. Clinical and neurohormonal disruptions are significantly related to NGAL levels. An increase in the level of NGAL is a result of cardiomyocytes in inadequate myocardium in both experimental and clinical HF studies [2]. In this study, plasma NGAL levels were higher in patients with HF, but statistically insignificant. However, troponin I, CKMB, and CRP levels were higher in STEMI patients with HF.

The prognostic impact of reduced GFR in chronic HF is known [2]. As is known, NGAL assessment plays an important role in the early detection of acute kidney injury [19]. In this study, renal functions were not evaluated in detail, except admission GFR and creatinine levels. Patients with creatinine > 1.4 mg/dl on admission were excluded. There are several reports in the literature, which suggest an increase in serum NGAL levels after coronary angiography and PCI due to contrast material and activation of neutrophils by direct tissue damage in PCI [20–22]. These reports suggest that angiography-induced increase in serum NGAL level lasts at most 24 hours. As we had collected the venous blood samples on admission in the coronary care unit or in the emergency department, we think that we had excluded the confounding effect of angiography and contrast material on NGAL levels. Nevertheless, GFR values were significantly higher in patients with HF and death patients. Tubulointerstitial injury may also indicate kidney damage, even in the normal glomerular filtration. Increased tubular markers in HF patients, even if GFR was normal, were associated with a worse result [23]. In the present study, the increased plasma NGAL levels may be due to renal dysfunction in HF and cardiovascular mortality.

In this study, plasma NGAL levels were higher in patients who died than in patients with reduced LVEF, and we did not observe a significant difference in plasma NGAL level between the patients with reduced LVEF and preserved LVEF. Increased plasma levels of NGAL in patients with AMI in our study seem to be the result of a higher degree of inflammation in patients with AMI rather than the extent of myocardial ischemia or severity of coronary atherosclerosis. In conclusion, our results show that the plasma NGAL level is significantly higher in died patients with STEMI. This finding is important because the plasma NGAL levels may predict the cardiovascular mortality in patients with STEMI. We believe that these findings may pave the way for further studies searching the role of NGAL in STEMI.

## 5. Limitations

The limitations of our study are as follows: first, it is single-centered and has a relatively small sample size, and secondly, NGAL levels were measured only at the time of admission and evaluated once.

## 6. Conclusion

According to the data we obtained, serum NGAL level measured at the time of admission in STEMI patients was strongly associated with mortality and is an indication that this may be used as a risk marker in STEMI patients.

## Figures and Tables

**Table 1:** Clinical and biochemical characteristics in patients with STEMI.

	*n* = 68

Female sex, *n* (%)	12 (17.6)
Age, years	61.51 ± 14.72
DM, *n* (%)	12 (17.6)
HT, *n* (%)	29 (42.6)
DL, *n* (%)	8 (11.8)
Smoking, *n* (%)	51 (75)
Family history of CAD, *n* (%)	9 (13.4)
Admission time (hours)	4.61 ± 2.96
Admission SBP (mmHg)	114.93 ± 22.58
Anterior wall MI, *n* (%)	25 (36.8)
Inferior wall MI, *n* (%)	33 (48.5)
Posterolateral wall MI, *n* (%)	10 (14.7)
Primer PCI, *n* (%)	40 (58.8)
Fibrinolytic administration, *n* (%)	30 (44.1)
NGAL (ng/ml)	190.08 (9.00–694.00)
Troponin I (ng/ml)	1.51 (0.10–25.00)
CKMB (ng/ml)	14.99 (1.63–380.00)
CRP (mg/dl)	0.53 (0.05–9.59)
Creatinine (mg/dl)	0.88 ± 0.19
GFR (ml/min)	101.47 ± 23.29
LDL (mg/dl)	124.35 ± 34.70
HDL (mg/dl)	36.65 ± 9.04
Haemoglobin (mg/dl)	14.83 ± 2.27
Leukocytes (K/uL)	12.27 ± 0.45
Platelets (K/uL)	240 (72–485)
Neutrophils (K/uL)	8.99 ± 4.07
Lymphocytes (K/uL)	2.21 ± 1.39

**Table 2:** Angiographic, clinical, and biochemical properties of patients in preserved EF and reduced EF groups.

	Preserved LVEF, *n* = 34	Reduced LVEF, *n* = 34	*p* value
Female sex, *n* (%)	6 (17.6)	6 (17.6)	0.62
Age, years	56.24 ± 13.57	66.79 ± 14.09	**0.002**
DM, *n* (%)	7 (20.6)	5 (14.7)	0.37
HT, *n* (%)	12 (35.3)	17 (50)	0.16
DL, *n* (%)	4 (11.8)	4 (11.8)	0.64
Smoking, *n* (%)	24 (70.6)	27 (79.4)	0.28
Family history of CAD, *n* (%)	6 (17.6)	3 (9.1)	0.25
Admission time (hours)	3.76 ± 2.84	5.45 ± 2.88	**0.01**
Admission SBP (mmHg)	122.79 ± 19.89	107.06 ± 22.63	**0.003**
Myocardial infarction localization			
Anterior wall, *n* (%)	2 (5.9)	23 (67.6)	**<0.001**
Inferior wall, *n* (%)	26 (76.5)	7 (20.6)	
Posterior wall, *n* (%)	6 (17.6)	4 (11.8)	
Primer PCI, *n* (%)	18 (52.9)	22 (64.7)	0.23
Fibrinolytic administration, *n* (%)	15 (44.1)	15 (44.1)	0.59
NGAL (ng/ml)	124 (9–382)	184 (21–694)	0.07
Troponin I (ng/ml)	0.92 (0.13–25.00)	3.77 (0.10–25.00)	**0.008**
CKMB (ng/ml)	6.60 (1.63–284.00)	29.92 (1.81–380.00)	**0.002**
CRP (mg/dl)	0.27 (0.05–5.53)	1.38 (0.44–9.59)	**<0.001**
Creatinine (mg/dl)	0.81 ± 0.15	0.96 ± 0.21	**0.001**
GFR (ml/min)	111.76 ± 18.64	91.17 ± 23.14	**<0.001**
LDL (mg/dl)	115.52 ± 37.28	133.18 ± 29.90	**0.03**
HDL (mg/dl)	34.69 ± 9.11	38.61 ± 8.67	0.07
Haemoglobin (mg/dl)	14.68 ± 2.19	14.98 ± 2.37	0.60
Leukocytes (K/uL)	11.53 ± 3.72	13.05 ± 5.05	0.16
Platelets (K/uL)	241 (72–428)	228 (80–485)	0.37
Neutrophils (K/uL)	8.07 ± 3.45	9.97 ± 4.48	**0.05**
Lymphocytes (K/uL)	2.26 ± 1.39	2.16 ± 1.40	0.77
Neutrophil-to-lymphocyte ratio	3.86 (0.56–13.94)	5.08 (0.88–19.90)	0.28

Significant *p* values are in bold. CAD, coronary artery disease; CKMB, creatine kinase MB; CRP, C-reactive protein; DL, dyslipidemia; DM, diabetes mellitus; GFR, glomerular filtration rate; HDL, high-density lipoprotein; HT, hypertension; LDL, low-density lipoprotein; LVEF, left ventricular ejection fraction; MI, myocardial infarction; NGAL, neutrophil gelatinase-associated lipocalin; PCI, percutaneous coronary intervention; SBP, systolic blood pressure.

**Table 3:** Logistic regression analysis with reduced EF as the dependent variable.

Independent variables	*β* ± SE	Wald	*p* value
NGAL	0.003 ± 0.002	1.285	0.25
Age	0.054 ± 0.022	6.139	**0.01**
Hypertension	0.345 ± 0.655	0.277	0.59
Diabetes mellitus	−0.842 ± 0.797	1.116	0.29
Dyslipidemia	0.174 ± 0.971	0.032	0.85
Smoking	0.631 ± 0.848	0.554	0.45
Sex	−0.352 ± 0.950	0.138	0.71
Troponin	0.073 ± 0.034	4.681	**0.03**

Significant *p* values are in bold. *β* ± SE, beta ± standard error; NGAL, neutrophil gelatinase-associated lipocalin.

**Table 4:** Angiographic, clinical, and biochemical properties of patients in death and survived groups.

	Death, *n* = 14	Survived, *n* = 54	*p* value
Female sex, *n* (%)	5 (35.7)	7 (13)	0.06
Age, years	73.36 ± 10.59	58.44 ± 14.14	**<0.001**
DM, *n* (%)	2 (14.3)	10 (18.5)	0.53
HT, *n* (%)	9 (64.3)	20 (37)	0.06
DL, *n* (%)	1 (7.1)	7 (%13)	0.47
Smoking, *n* (%)	9 (64.3)	42 (77.8)	0.23
Family history of CAD, *n* (%)	0 (0)	9 (16.7)	0.12
Admission time (hours)	6.35 ± 2.87	4.15 ± 2.84	**0.01**
Admission SBP (mmHg)	103.57 ± 27.90	117.87 ± 20.27	**0.03**
Myocardial infarction localization			
Anterior wall MI, *n* (%)	9 (64.3)	16 (29.6)	**0.04**
Inferior wall MI, *n* (%)	3 (21.4)	30 (55.6)	
Posterolateral wall MI, *n* (%)	2 (14.3)	8 (14.8)	
Primer PCI, *n* (%)	8 (57.1)	32 (59.3)	0.56
Fibrinolytic administration, *n* (%)	8 (40.7)	22 (57.1)	0.21
NGAL (ng/ml)	357 (71–694)	120 (9–513)	**<0.001**
Troponin I (ng/ml)	7.39 (0.30–25.00)	1.05 (0.1–25.00)	0.15
CKMB (ng/ml)	20.49 (3.05–300.00)	12.33 (1.63–380.00)	0.21
CRP (mg/dl)	0.71 (0,05–7.06)	0.52 (0.09–9.59)	0.83
Creatinine (mg/dl)	1.01 ± 0.21	0.85 ± 0.17	**0.007**
GFR (ml/min)	78.35 ± 22.53	107.46 ± 19.58	**<0.001**
LDL (mg/dl)	119.25 ± 28.11	125.67 ± 36.32	0.54
HDL (mg/dl)	38.92 ± 10.30	36.06 ± 8.70	0.29
Haemoglobin (mg/dl)	13.70 ± 2.34	15.08 ± 2.19	0.06
Leukocytes (K/uL)	13.06 ± 4.57	12.09 ± 4.45	0.49
Platelets (K/uL)	262 (80–428)	239 (72–485)	0.66
Neutrophils (K/uL)	10.15 ± 4.20	8.73 ± 4.03	0.27
Lymphocytes (K/uL)	1.97 ± 0.82	2.26 ± 1.48	0.50
Neutrophil-to-lymphocyte ratio	5.08 (2.04–10.74)	4.44 (0.56–19.90)	0.38

Significant *p* values are in bold. CAD, coronary artery disease; CKMB, creatine kinase MB; CRP, C-reactive protein; DL, dyslipidemia; DM, diabetes mellitus; GFR, glomerular filtration rate; HDL, high-density lipoprotein; HT, hypertension; LDL, low-density lipoprotein; MI, myocardial infarction; NGAL, neutrophil gelatinase-associated lipocalin; PCI, percutaneous coronary intervention; SBP, systolic blood pressure.

**Table 5:** Logistic regression analysis with cardiovascular mortality as the dependent variable.

Independent variables	*β* ± SE	Wald	*p* value
NGAL	0.017 ± 0.007	5.590	**0.01**
Age	0.078 ± 0.047	2.813	0.09
Hypertension	0.466 ± 1.275	0.134	0.71
Diabetes mellitus	−0.428 ± 1.606	0.071	0.79
Dyslipidemia	−2.603 ± 1.944	1.794	0.18
Smoking	0.357 ± 1.855	0.037	0.84
Sex	3.415 ± 2.290	2.224	0.13
Heart failure	5.110 ± 2.444	4.372	**0.03**
Troponin	−0.139 ± 0.97	2.062	0.15

Significant *p* values are in bold. *β* ± SE, beta ± standard error; NGAL, neutrophil gelatinase-associated lipocalin.

## Data Availability

The data used to support the findings of this study are available from the corresponding author upon reasonable request.
